# Supplementary material for: Structural basis of trans-synaptic interactions between PTPδ and SALMs for inducing synapse formation
Source: Nat Commun. 2018 Jan 18;9:269. doi: 10.1038/s41467-017-02417-z (PMC5773591; doi:10.1038/s41467-017-02417-z)
Supplement: Supplementary file 1 — Supplementary Information [file 41467_2017_2417_MOESM1_ESM.pdf]

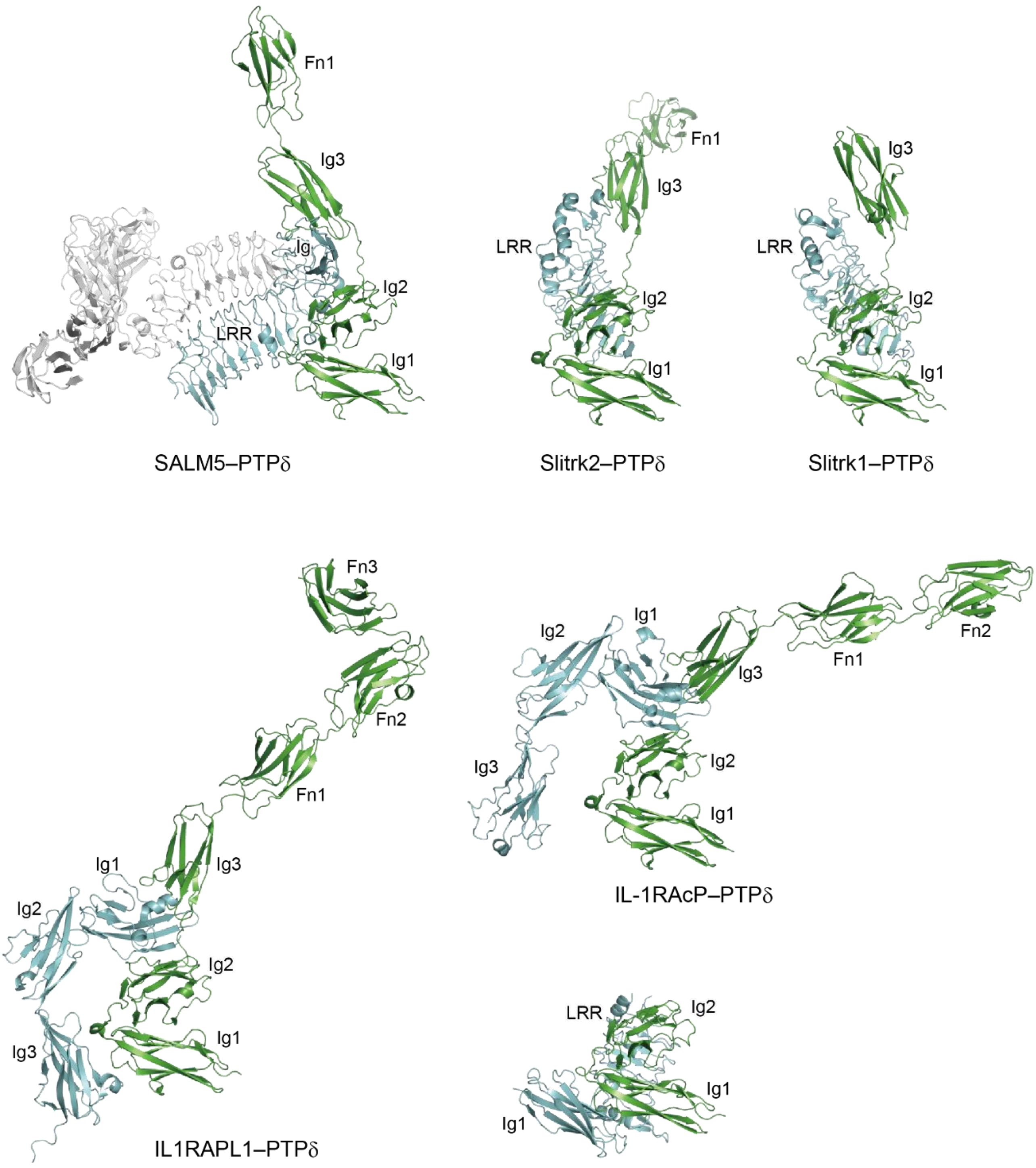

Supplementary Figure 1 Structures of complexes between type-IIa RPTPs and postsynaptic organizers.

Type-IIa RPTPs and postsynaptic organizers are colored in green and cyan, respectively.

Ligand : SALM5   Analyte : PTPδ splicing variant

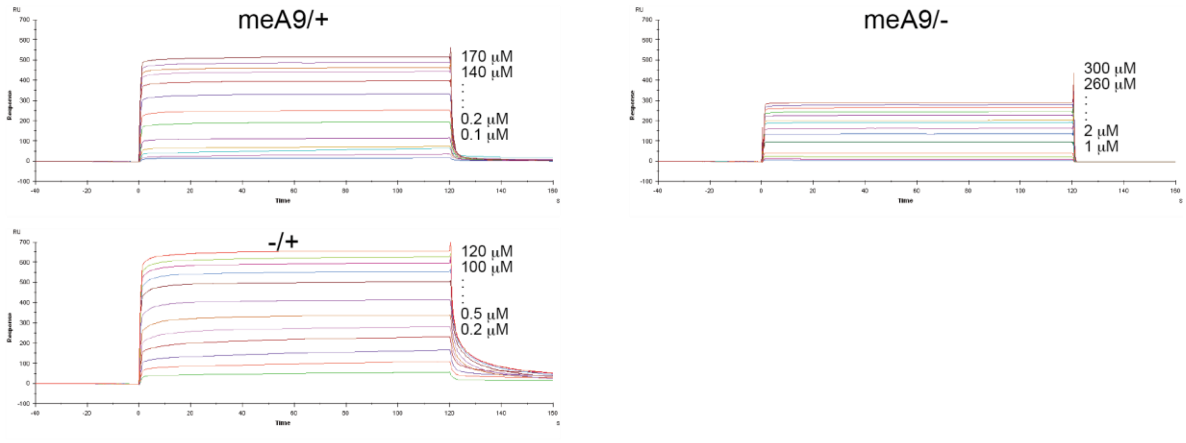

Supplementary Figure 2 SPR sensorgrams for interaction analyses between SALM5 and PTPδ splicing variants.

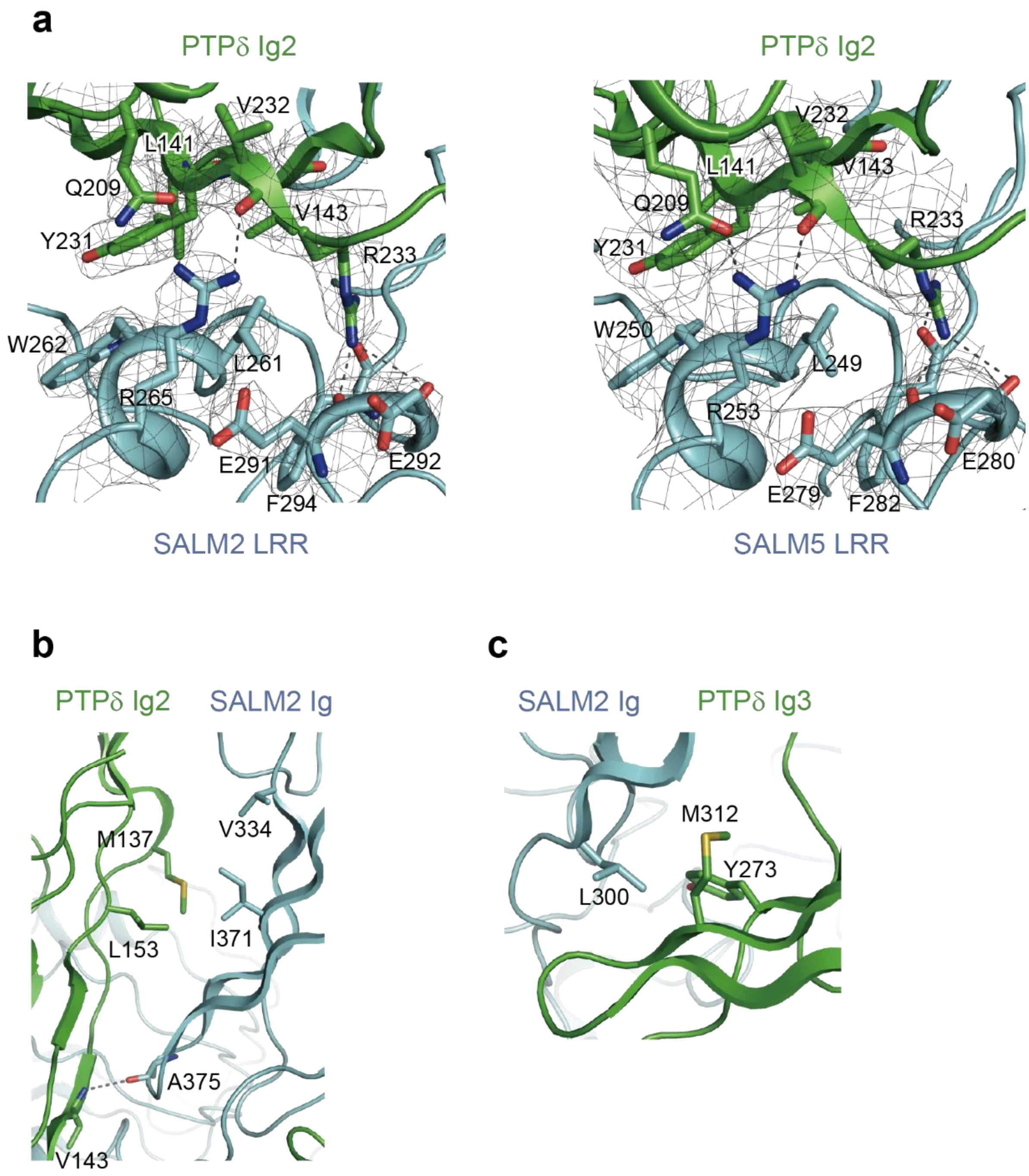

Supplementary Figure 3　Interface between PTPδ and SALM2.

Hydrogen bonds are indicated as dotted lines.

(a) Interface between PTPδ Ig2 and SALM2 LRR. The electron density of the area around this interface is shown as a $2F_o$–F_c map contoured at 1.3 σ level. For comparison, the electron density of the area around the interface between PTPδ Ig2 and SALM5 LRR is also shown ($2F_o$–F_c map contoured at 1.3 σ level).

(b) Interface between PTPδ Ig2 and SALM2 Ig.

(c) Interface between PTPδ Ig3 and SALM2 Ig.

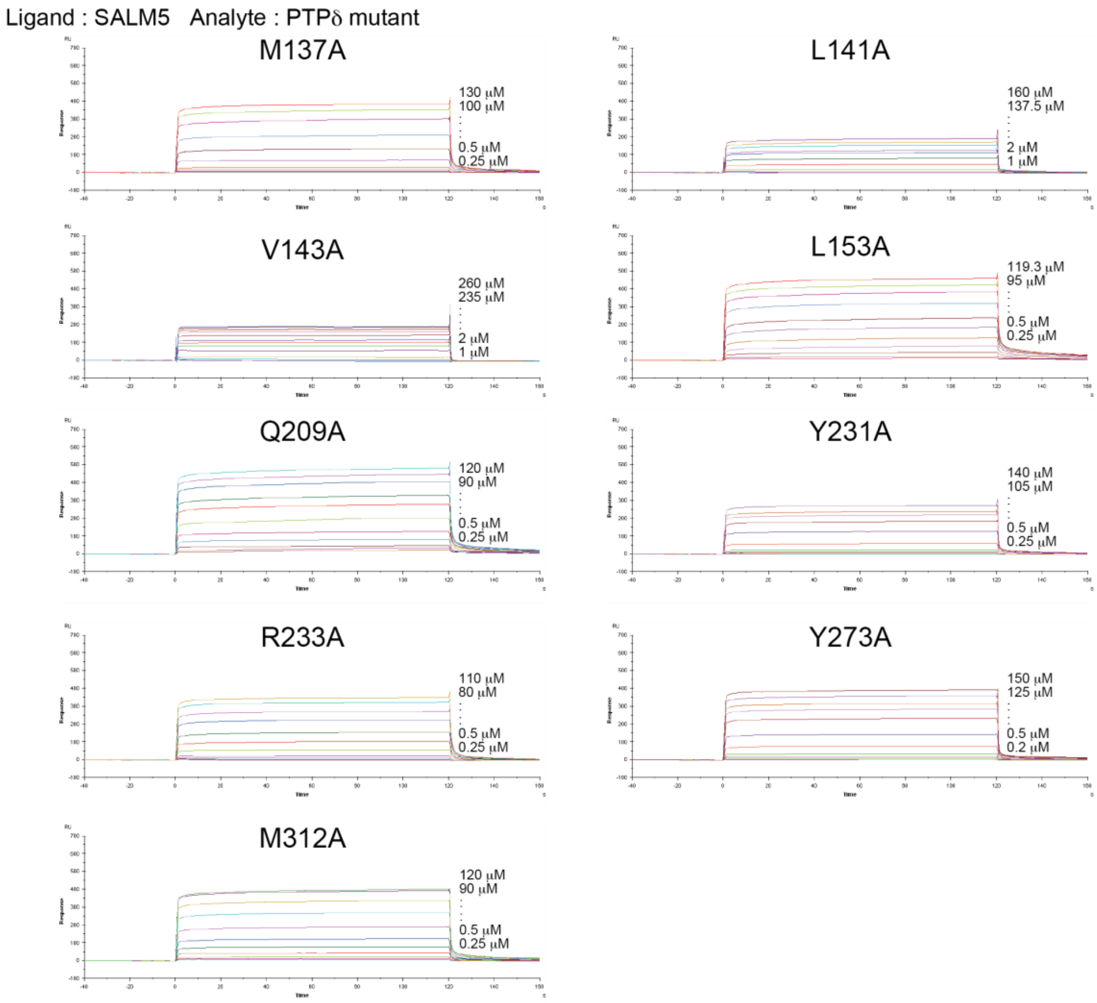

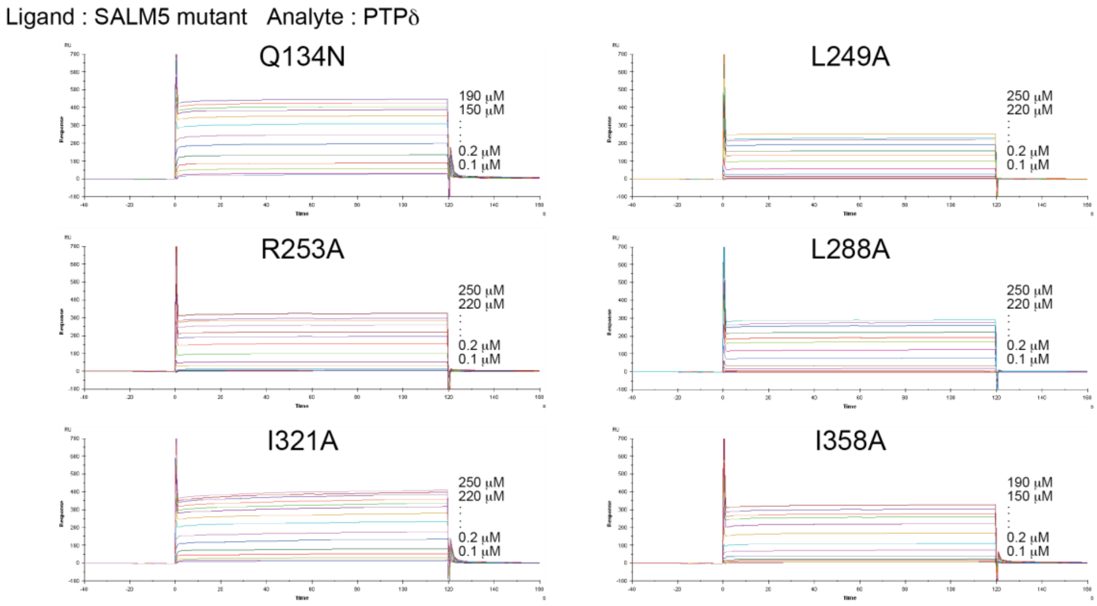

Supplementary Figure 4 SPR sensorgrams for interaction analyses between mutant PTPδ and SALM5 or between PTPδ and mutant SALM5.

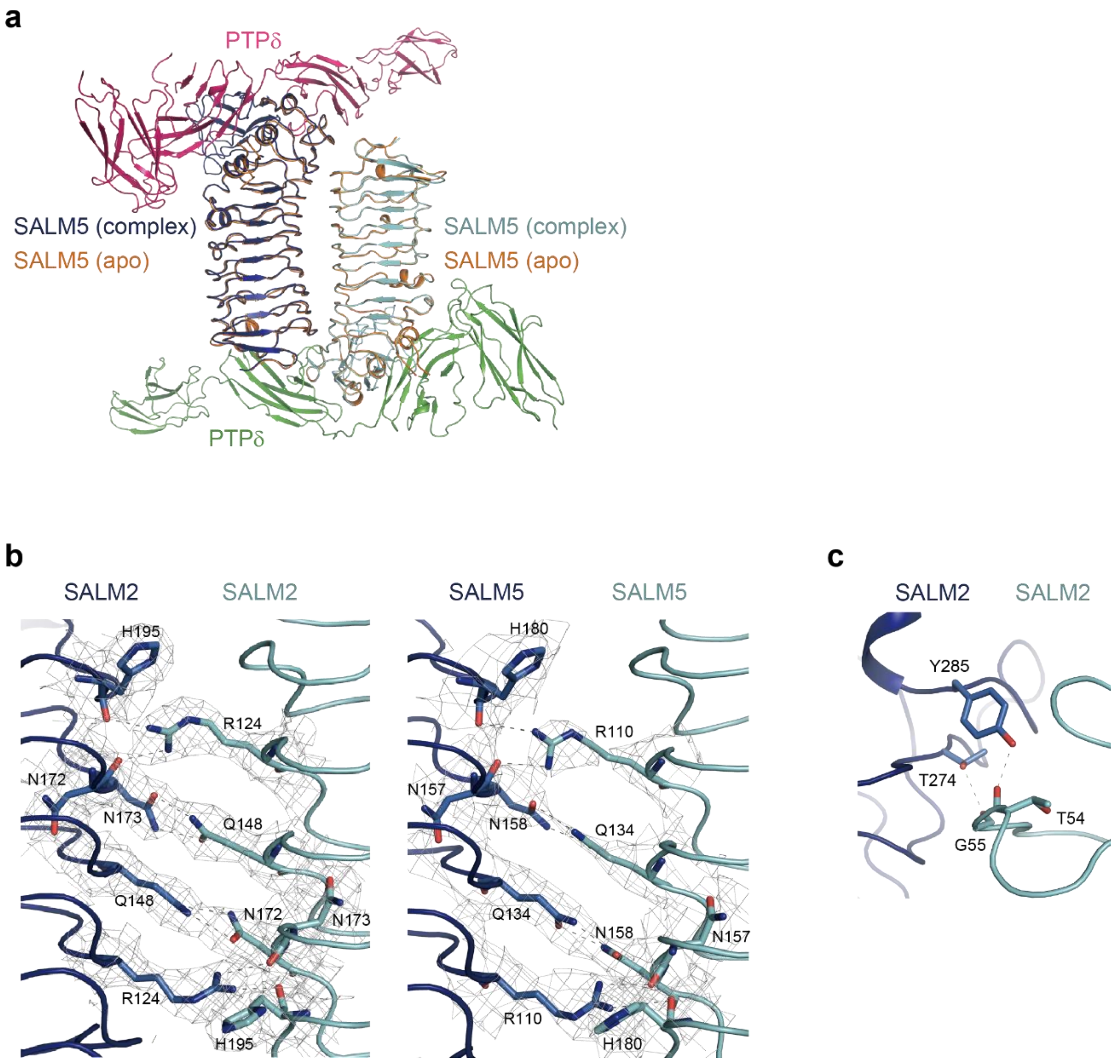

Supplementary Figure 5 LRR-mediated dimer of SALM2 and SALM5.

(a) Superposition of SALM5 dimers from PTPδ–SALM5 complex and apo-SALM5.

(b) Interactions in the central region of the SALM2 dimer interface. Hydrogen bonds are indicated as dotted lines. The electron density of the area around this interface is shown as a $2F_o–F_c$ map contoured at 1.1 σ level. For comparison, the electron density of the area around the central region of the SALM5 dimer interface is also shown ($2F_o–F_c$ map contoured at 1.1 σ level).

(c) Interactions in the peripheral region of the SALM2 dimer interface. Hydrogen bonds are indicated as dotted lines.

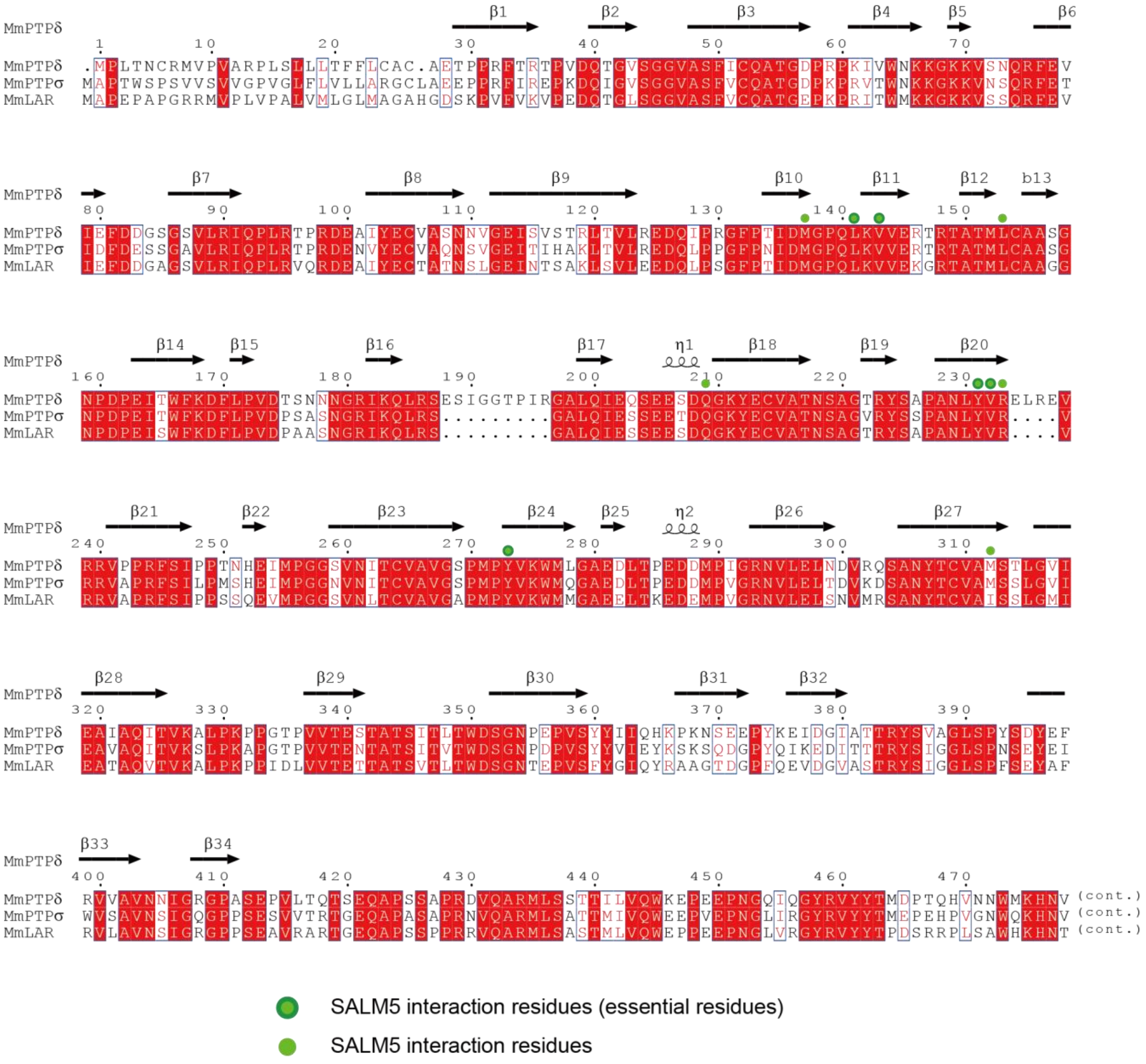

Supplementary Figure 6 Amino acid sequence alignment of type-IIa RPTPs.

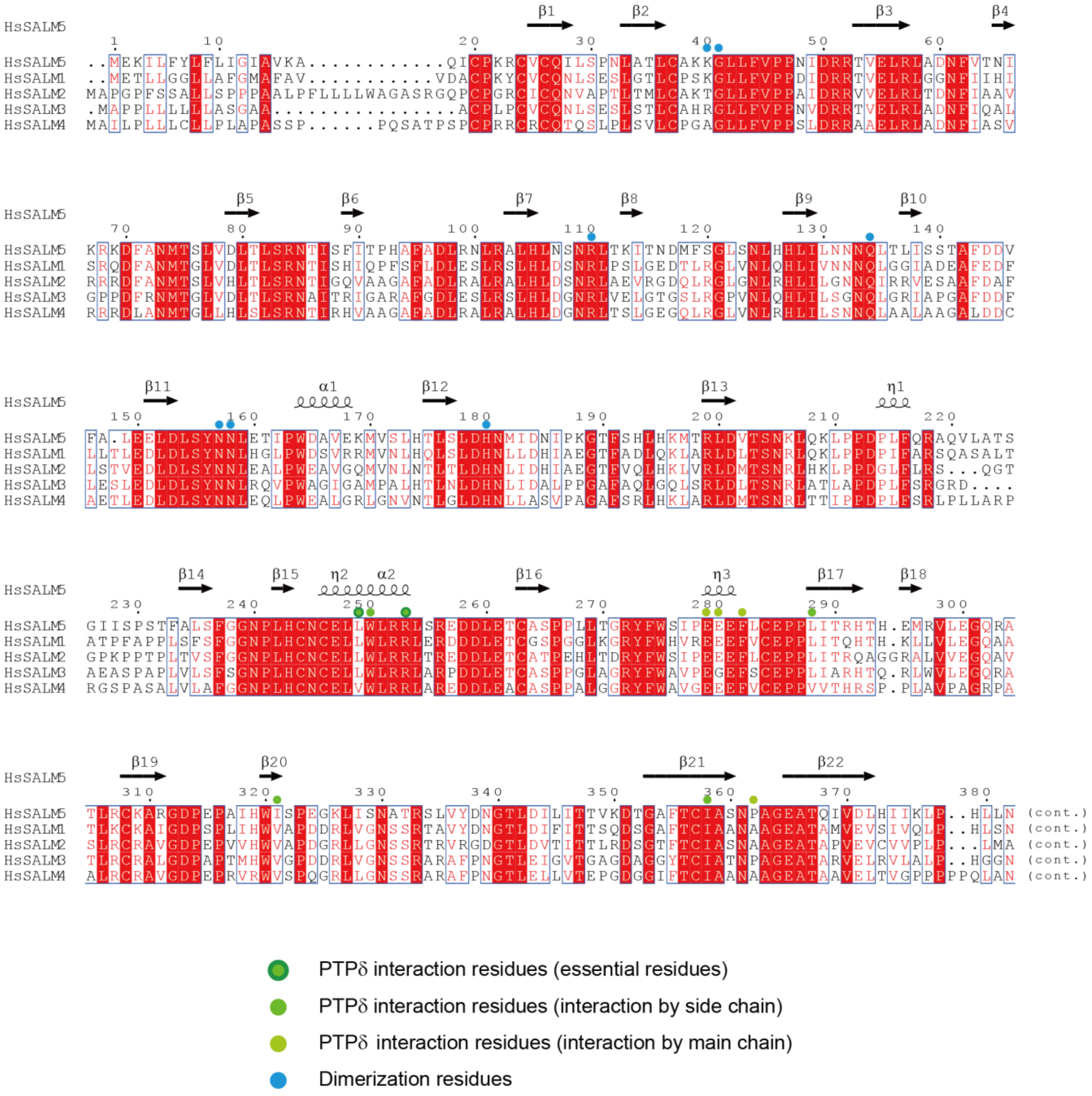

Supplementary Figure 7   Amino acid sequence alignment of SALMs.

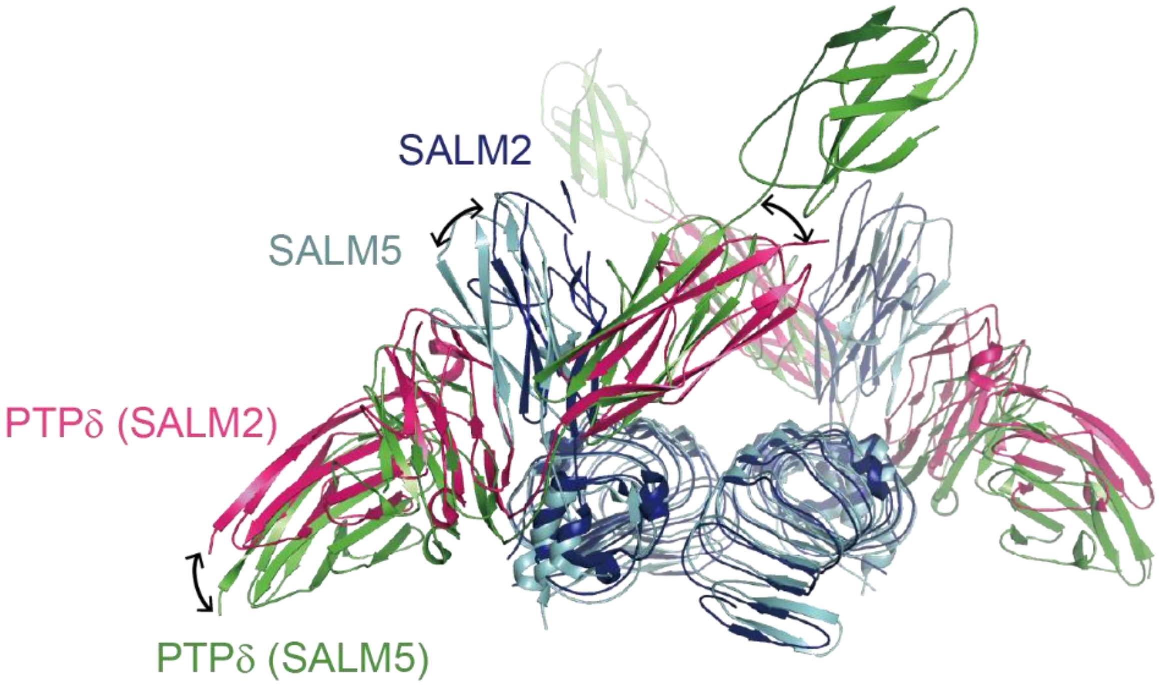

Supplementary Figure 8 Superposition of PTPδ–SALM5 and PTPδ–SALM2 complexes.
PTPδ–SALM5 and PTPδ–SALM2 complexes are superposed by the LRR domains of SALM dimers.

	SALM2	SALM5
PTPδ Ig2–SALM LRR	Leu261	Leu249
	Trp262	Trp250
	Arg265	Arg253
	Glu291	Glu279
	Glu292	Glu280
	Phe294	Phe282
PTPδ Ig2–SALM Ig	Val334	Ile321
	Ile371	Ile358
	Ala375	Pro362
PTPδ Ig3–SALM Ig	Leu300	Leu288
SALM LRR–SALM LRR (dimerization)	Thr54	Lys40
	Gly55	Gly41
	Arg124	Arg110
	Gln148	Gln134
	Asn172	Asn157
	Asn173	Asn158
	His195	His180
	Thr274	Thr262
	Tyr285	Tyr273

Supplementary Table 1 Conservation of the amino acid residues involved in the interaction with PTPδ or homodimerization of SALMs between SALM2 and SALM5.
